# Effects of Sleeve Gastrectomy vs. Roux-en-Y Gastric Bypass on Eating Behavior and Sweet Taste Perception in Subjects with Obesity

**DOI:** 10.3390/nu10010018

**Published:** 2017-12-24

**Authors:** Katie Nance, J. Christopher Eagon, Samuel Klein, Marta Yanina Pepino

**Affiliations:** 1 Department of Food Science and Human Nutrition, College of Agricultural, Consumer and Environmental Sciences, University of Illinois, Urbana-Champaign, Champaign, IL 61801, USA; maryn2@illinois.edu; 2 Department of Surgery, Washington University School of Medicine, St. Louis, MO 63110, USA; j.chris.eagon@wustl.edu; 3 Center for Human Nutrition, Washington University School of Medicine, St. Louis, MO 63110, USA; sklein@wustl.edu

**Keywords:** taste, eating behavior, gastric bypass, sleeve gastrectomy, bariatric surgery

## Abstract

The goal of this study was to test the hypothesis that weight loss induced by Roux-en-Y gastric bypass (RYGB) has greater effects on taste perception and eating behavior than comparable weight loss induced by sleeve gastrectomy (SG). We evaluated the following outcomes in 31 subjects both before and after ~20% weight loss induced by RYGB (*n* = 23) or SG (*n* = 8): (1) sweet, savory, and salty taste sensitivity; (2) the most preferred concentrations of sucrose and monosodium glutamate; (3) sweetness palatability, by using validated sensory testing techniques; and (4) eating behavior, by using the Food Craving Inventory and the Dutch Eating Behavior Questionnaire. We found that neither RYGB nor SG affected sweetness or saltiness sensitivity. However, weight loss induced by either RYGB or SG caused the same decrease in: (1) frequency of cravings for foods; (2) influence of emotions and external food cues on eating behavior; and (3) shifted sweetness palatability from pleasant to unpleasant when repetitively tasting sucrose (all *p*-values ≤ 0.01). Therefore, when matched on weight loss, SG and RYGB cause the same beneficial effects on key factors involved in the regulation of eating behavior and hedonic component of taste perception.

## 1. Introduction

Bariatric surgery procedures provide the most successful long-term treatment for obesity and its related comorbidities [1]. Among the different surgical procedures, sleeve gastrectomy (SG) and Roux-en-Y gastric bypass (RYGB) are the most commonly performed procedures worldwide [2]. Both procedures decrease gastric volume and result in marked weight loss, but, unlike SG, in which the continuity of the intestine remains intact, RYGB reroutes the intestine so that nutrients bypass most of the stomach and the duodenum and flow directly into the jejunum. Patients who have undergone RYGB surgery tend to lose more weight than those who have undergone SG [3,4,5,6,7], at least within the first year follow up. 

The precise mechanisms responsible for this difference in weight loss between procedures is unknown, but we hypothesize that it is related, at least in part, to surgically dependent changes in taste perception that subsequently influence eating behavior and can steer patients toward healthier food choices. Taste perception is the psychological factor of gustation and involves two major components: the sensory-discriminative component and the hedonic component [8]. The sensory-discriminative component encompasses taste quality (i.e., sweet, salty, bitter, sour, savory/umami) and taste sensitivity. Taste sensitivity ranges from responses at the taste detection threshold (the lowest concentration of a taste stimulus that can be detected in solution) to those well above the detection threshold, which more closely relates to concentrations of taste stimuli in food [8]. The hedonic component refers to the palatability of the stimulus, which directly influences food reward [8].

Patients who have undergone RYGB surgery decrease the proportion of their daily calorie intake from sweetened and calorie-dense foods [9,10,11]. While the specific advice given to patients to avoid sweetened and calorie-dense food to prevent symptoms of dumping syndrome may contribute to the reduction in the consumption of these foods, data from several studies support the hypothesis that these changes in eating behavior are mediated, at least in part, by surgery-induced effects in brain reward. RYGB-induced weight loss: (1) selectively reduced neural activation in reward-related brain areas when looking at pictures of highly palatable foods [12,13,14]; (2) decreased the motivation to work for a candy [15], and (3) lowered hedonic hunger [16]. Also consistent with this hypothesis, and convergent with data from preclinical studies [17,18,19], we have recently found that RYGB has weight loss-independent effects on the hedonic dimension of sweet taste perception in people, shifting palatability from pleasant to unpleasant after repetitively tasting sucrose [20]. Furthermore, we found that the dislike patients experience when repetitively tasting something sweet after RYGB is not due to an enhanced perception of sweetness intensity. By using a battery of well-validated sensory techniques, we found that the sensory-discriminative component of taste perception remains remarkably unchanged after RYGB [20].

While RYGB remains a heavily studied procedure, the current literature concerning SG and taste perception is growing but still highly nascent. Although patients frequently report changes in taste perception following SG [21,22,23] and findings from survey studies suggest that SG modifies sweet taste perception [24,25], few studies have carefully measured changes in taste perception in these patients using validated sensory techniques [26]. 

The primary goal of this study was to determine the effect of SG on taste perception by using a validated battery of sensory evaluation techniques, and to test the hypothesis that weight loss induced by RYGB confers greater effects on taste perception and eating behavior than matched weight loss induced by SG. 

## 2. Materials and Methods

### 2.1. Subjects

Thirty-one (31) subjects who were to undergo either RYGB (*n* = 23) or SG (*n* = 8) at the Barnes-Jewish hospital in St. Louis, Missouri, USA participated in this study. The initial recruitment of subjects involved reviewing medical records and in-person interviews conducted at the Bariatric Surgery Clinic. We excluded patients who: were on medications that could alter taste, had undergone a previous gastrointestinal surgery, smoked cigarettes, had a diagnosis of diabetes or were taking medicine to treat diabetes, showed signs of oral disease, exhibited a history of chronic rhinitis, had irritable bowel syndrome, or were experiencing severe organ dysfunction. Data from a subsample of these subjects have been reported previously [20]. Patients provided written informed consent, which was approved by the Washington University Institutional Review Board (IRB ID#201101965).

### 2.2. Experimental Procedures

Subjects were asked to fast for 12 h overnight at home prior to being tested in the Clinical Research Unit at the Washington University School of Medicine. To diminish the risk of sensory fatigue, sensory testing took place over three separate 2-h visits, the order of which was randomized for each subject. Visits were scheduled at least one day apart. At each visit, subjects were tested on one of the three following categories: (1) Monosodium Glutamate (MSG), (2) Sucrose, or (3) NaCl/Glucose. Detection thresholds were always performed first to avoid exposing receptors to high concentrations early on, followed by suprathresholds and preferences. The sweet taste palatability test was always performed at the end of the MSG day (i.e., the day without glucose or sucrose testing). For all sensory procedures, 10 mL of each solution were tasted using the swish-and-spit method. After subjects lost ~20% of their initial weight, the same experimental procedures were distributed over 3 separate 2 h visits and evaluated in the same manner as described for pre-surgery.

#### 2.2.1. Sensory-Discriminative Component of Taste Perception

1. Detection Thresholds: Sucrose, glucose, NaCl, and MSG (monosodium glutamate, for savory/umami quality) detection thresholds were determined by utilizing a two-alternative, forced-choice staircase procedure [27].
2. Suprathreshold: Subjects were trained on the use of the general labelled magnitude scale (gLMS) before we measured perceived intensities [28]. Two trials consisting of 4 ascending concentrations of each stimulus (sucrose, glucose, NaCl, MSG) with the first “concentration” being water were presented to the subjects. All four concentrations were presented in random order without repeat. Subjects rated the perceived intensity of the stimulus using the gLMS and we used the mean intensities of the two trials at each concentration for each stimulus to evaluate subjects’ taste intensity perception.

#### 2.2.2. Hedonic Component of Taste Perception

1. Preference Tests. We measured preferred concentrations of sucrose and MSG by using the Monell forced-choice, paired comparison tracking procedure [29]. Subjects were initially presented with two mid-range concentrations (180 vs. 700 mmol/L for sucrose or 11 vs. 37 mmol/L for MSG). They tasted the concentrations without swallowing and chose which solution they preferred. This procedure continued until they chose one concentration over concentrations that were both higher and lower, or the highest or lowest concentrations were chosen twice in a row. We presented the pairs in reverse order for the second trial. Subjects were allowed a 1 min interval between each pair, during which they rinsed their palette with water [27,29].
2. Sweet Taste Palatability Test. Subjects tasted, without swallowing, a series of 10 samples containing 10 mL of 700 mmol/L (24% *w*/*v*) sucrose solution. They were asked to taste each sample for 10 s and received a new sample every two minutes with no interstimulus rinse. Immediately after tasting each sample, subjects rated the following two questions: “How pleasant was the taste?”, and “How strong is your desire for a different taste?” using a hedonic gLMS [28].

### 2.3. Taste Stimuli 

For detection threshold testing, sucrose, glucose, NaCl (all from Sigma-Aldrich, Inc., St. Louis, MO, USA) and MSG (USB Corp., Cleveland, OH, USA) concentrations ranging from 1 to 1 × 10^−4^ M were prepared in quarter-log dilution steps. For suprathreshold taste intensity perception, we used 0, 90, 360, and 1050 mmol/L sucrose solutions, 0, 320, 560 and 1000 mmol/L glucose solutions, 0, 56, 180, and 560 mmol/L NaCl solutions, and 0, 20, 60, and 180 mmol/L MSG solutions. All solutions were prepared using deionized water and presented at room temperature (22 °C). For determining preferences, we used 90, 180, 350, 700, and 1005 mmol/L sucrose and 18, 32, 56, 100, and 180 mmol/L MSG. Previous studies have shown that MSG decreases palatability of room temperature water but increases palatability of warm foods, such as soup [30]. Therefore, we warmed the MSG solutions in water to 40 °C and stored them in thermal containers until testing time.

### 2.4. Eating Behavior

Subjects completed the Dutch Eating Behavior Questionnaire (DEBQ) [31] and the Food Craving Inventory (FCI) [32] both pre- and post-surgery. The DEBQ is a widely used validated questionnaire that measures responses to three behavioral aspects related to food consumption: (I) emotional, which involves eating in response to negative feelings such as depression or boredom; (II) external, or the response to the sight or the smell of food; and (III) restraint, which encompasses an inclination to consciously restrict food intake to control body weight [31]. The FCI is a self-report questionnaire where subjects rate their frequency of cravings for 48 food items during the previous month. The scores on this questionnaire provide a validated measure of the frequency of overall food cravings as well as cravings for 4 specific types of foods (sweets, starches, high fats, and fast-food fats) [32]. For both scales, subjects rate the frequency on which they engage in these behaviors using a 5 point Likert scale ranging from 1 (never) to 5 (very often/always). 

### 2.5. Surgical Procedures 

Bariatric surgeries were performed using standard laparoscopic approaches. The SG procedure involved dividing the gastrocolic ligament, initiating the gastrectomy 6 cm proximal to the pylorus along the greater curve, and creating the sleeve along the lesser curve over a 40 French Bougie. The RYGB procedure involved creating a small (~20 mL) proximal gastric pouch and a stapled gastrojejunostomy. A 75–150 cm Roux-Y limb was constructed by transecting the jejunum 30 cm distal to the ligament of Treitz and performing a stapled jejunojejunostomy at this site [20].

### 2.6. Diet Management after Surgery

A study dietitian consulted with subjects weekly, either over the phone or in person. The dietitian provided standard weight management behavioral education, monitored weight loss, reviewed dietary intake, and adjusted diets in order to meet the targeted body weight loss goal of ~20% within 4–6 months post-surgery. For both groups, the first week after surgery involved a liquids-only diet. Progression to regular foods occurred 2–4 weeks following surgery with the limit of 1000–1200 kcal/day and 1 g protein/kg body weight/day. 

### 2.7. Statistical Analyses 

Two-way ANOVAs with group (SG vs. RYGB) as the categorical factor and time (pre-surgery vs. post-surgery), and trials (1–10) (when repetitively tasting sucrose), as the within effects factor(s) were used to assess if type of bariatric surgery had a significant effect on the study outcome measures. Detection thresholds and preferences for sucrose and MSG were positively skewed and thus transformed logarithmically to approximate a normal distribution. Desire for a different taste when repetitively tasting sucrose and perceived intensity of taste stimuli above-threshold were also positively skewed and required square root transformations to approximate a normal distribution. Skewed data are reported in the tables as mean ± semi-interquartile range. Due to technical problems, three (out of the 23) subjects in the RYGB group did not complete suprathreshold intensity perception for glucose. Analyses were performed with Statistica versions 13.0 and 13.3 (StatSoft Inc., Tulsa, OK, USA). We adjusted for multiple testing using the false discovery rate (FDR; Benjamini-Hochberg) [33] and a *p*-value ≤ 0.01 determined statistical significance.

### 2.8. Power Analysis

Differences in taste detection thresholds were the primary outcome measures of this study. Based on reproducibility data from a previous study [34], we estimated that 8 subjects in each surgery group would be needed to detect a 70% difference in taste detection thresholds between the RYGB and the SG group with a *β*-value of 0.20 (i.e., 80% power) and an *α*-value of 0.05. This proposed difference was a reasonable expectation based on data from other taste perception studies conducted in people, which found obesity is associated with a 100% increase in detection thresholds for MSG [27].

## 3. Results

### 3.1. Demographics of Subjects

There were no significant differences in body mass index (BMI) pre- and post-surgery or in characteristics that have been previously associated with sweetness preferences, such as age and race between groups. As expected, RYGB subjects tended to achieve ~20% weight loss quicker than SG patients did (*p* = 0.02); however, this trend failed to pass the false discovery rate threshold (Table 1).

### 3.2. Sensory-Discriminative Component of Taste Perception

**Figure 1:**
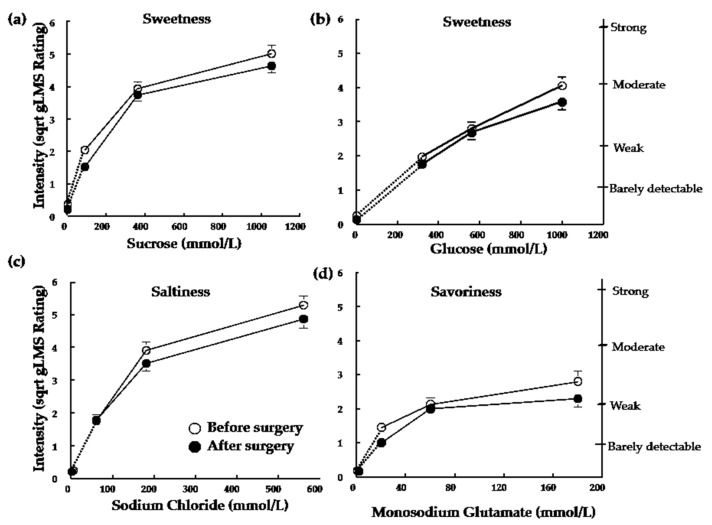
Perceived sweetness of increasing concentrations of (**a**) sucrose and (**b**) glucose, saltiness of increasing concentrations of (**c**) sodium chloride, and savoriness of increasing concentrations of (**d**) monosodium glutamate before (open symbols) and after (closed symbols) ~20% weight loss induced by bariatric surgery. Roux-en-Y Gastric Bypass and Sleeve Gastrectomy groups were combined because there was no main effect of group, nor interaction. Data are mean values ± standard error of the mean.

Taste detection thresholds after surgery-induced weight loss were not significantly different than those measured before surgery for subjects in either the SG or RYGB group (all *p* > 0.23; Table 2). There were no significant effects of weight-loss surgery on perceived sweetness of sucrose or glucose or the saltiness of NaCl (all *p* > 0.05; Figure 1a–c). Subjects perceived MSG as less intense following both RYGB and SG (*F*_(1,29)_ = 6.75; *p* = 0.02; Figure 1d). However, this trend failed to pass the false discovery rate threshold. 

### 3.3. Hedonic Component of Taste Perception 

#### 3.3.1. Preferences

Subjects equally lowered their favorite sucrose concentrations after SG and RYGB (Supplementary Figure S1). Two-way ANOVA revealed a main effect of time (before surgery vs. after surgery) (*F*_(1,29)_ = 5.17; *p* = 0.03). However, this trend failed to pass the false discovery rate threshold. There was no main effect of group (*p* = 0.26) nor interaction between group and time (*p* = 0.64). The most preferred MSG concentration did not change after surgery for either group (SG; before surgery: 58 ± 50 mm/L and after surgery: 58 ± 60 mm/L; RYGB before surgery: 102 ± 70 mm/L vs. after surgery: 55 ± 36 mm/L; *p* = 0.48).

#### 3.3.2. Sweet Taste Palatability 

**Figure 2:**
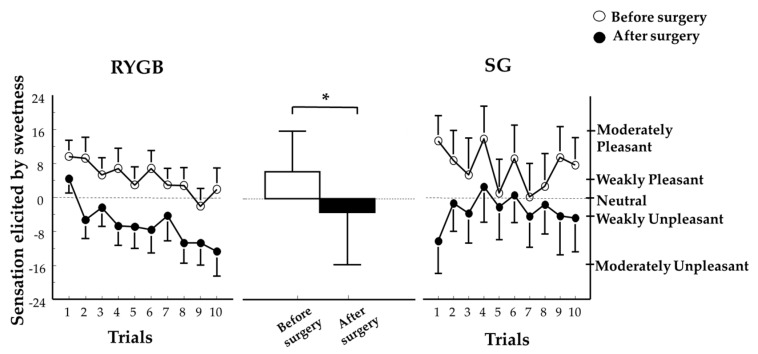
Hedonic value of sweetness when tasting an unswallowed sucrose solution across 10 trials before (open symbols, white bar) and after (closed symbols, black bar) ~20% weight loss induced by RYGB or SG. Two-way ANOVA revealed a main effect of time (before surgery vs. after surgery) (*F*_(1,29)_ = 7.5 ;* *p* = 0.01) RYGB and SG groups were combined because there was no main effect of group, nor interaction (all *p* values > 0.19). The right axis (illustrated for SG only but applicable for both groups) shows descriptors visualized by subjects when using the general labeled magnitude scale (gLMS). The left axis (illustrated for RYGB only but applicable for both groups) shows numbers corresponding to those descriptors on the scale. These numbers are not seen by subjects but experimenters receive them from the computer program. Data are mean values ± standard error of the mean RYGB, Roux-en-Y Gastric Bypass; SG, Sleeve Gastrectomy.

A two-way ANOVA revealed a main effect of time (*F*_(1,29)_ = 7.5; *p* = 0.01) with no other main effects nor interactions between groups and time (all *p* > 0.19). Sweet taste palatability during repetitive tasting of sucrose significantly shifted from pleasing to unpleasing following weight loss induced by either SG or RYGB (Figure 2). The desire to taste something different than sweet when sucrose was repetitively tasted increased with time equally in both groups and was unchanged from before surgery (*p* = 0.001; data not shown). 

### 3.4. Eating Behavior

SG and RYGB caused similar changes in eating behavior, with the exception of a trend for SG to cause a greater decrease in subjects’ frequency of cravings for carbohydrates than RYGB (*p* = 0.07; Table 2) and a trend for SG, but not RYGB, to cause an increase in restrained eating behavior (*p* = 0.06; Table 2). Both SG and RYGB caused a significant decrease in subjects’ frequency of cravings for high fat foods (−14 ± 5%), sweets (−26 ± 4%), and fast foods (−24 ± 4%) (all *p* < 0.005; Table 2). In addition, SG and RYGB caused similar decreases on the influence of emotions (−29 ± 4%) and external food cues on eating behavior (−26 ± 3%).

## 4. Discussion

The fundamental contribution of this study is the finding that weight loss induced by SG and RYGB causes comparable changes on eating behavior and similarly decreases the hedonic value of sweet taste. Weight loss induced by either SG or RYGB caused the same decrease in frequency of cravings for foods and influence of emotions and external food cues on eating behavior. Neither SG nor RYGB affected the sensory-discriminative dimension of taste perception. Detection thresholds for sucrose, glucose, NaCl, and MSG, as well as the perceived intensity of different concentrations of sucrose, glucose, or sodium chloride, were unchanged after surgery. 

Our finding of comparable effects between SG and RYGB on eating behavior extends our previous finding of comparable effects between RYGB and laparoscopic gastric banding (LAGB), a restrictive procedure where the anatomy of the stomach and intestine remains unchanged, on these same outcome variables [20]. Considering the radically different weight-loss procedures, these findings further suggest that attenuated food cravings and reduced emotional and external eating behavior are not due to anatomical and physiological changes associated with a particular surgical procedure, but are due to changes in dietary intake and weight loss following surgery. This interpretation is consistent with findings from previous studies that manipulate dietary intake in subjects who did not undergo bariatric surgery. For example, subjects who adhere to a very low-calorie diet for three months also reduced their cravings for foods, and the types of foods they restricted the most were the ones they craved the least [35,36]. 

Interestingly, in our previous study, we found that RYGB caused changes in the hedonic component of sweet taste perception whereas LAGB did not [20]; however, here we found that SG, similar to RYGB, decreased the pleasure elicited by sweet taste perception when repetitively tasting sucrose. This finding is consistent with data from rodent models of bariatric surgery that show that, despite remarkable differences in the anatomical rearrangements of SG and RYGB, both surgical procedures result in similar shifts toward consumption of less sugar and less calorically dense foods [37]. Although our data does not provide a mechanism underlying the comparable alteration in sweet taste palatability between SG and RYGB, it contributes to the literature by demonstrating that changes in sweet taste sensitivity is not one of those mechanisms. The present findings, in conjunction with data from a study showing no changes in sweet taste sensitivity after a 6-month dietary weight-loss intervention [38], suggest that sweet taste sensitivity is resistant to change following weight loss regardless of the manner in which the weight loss is achieved. Findings from a study in bariatric patients suggest that changes in satiety gut hormones, particularly glucagon-like peptide-1 (GLP-1) and peptide tyrosine-tyrosine YY (PYY), which are greatly enhanced post-prandially after both surgical procedures [39,40], could potentially be one of the underlying mechanisms of modified brain reward responses to food. By using well-validated behavioral and functional neuroimaging methods, Goldstone and collaborators found that the acute suppression of PYY and GLP-1 after a meal, by administration of a somatostatin analogue, increased food reward in patients after RYGB [41]. 

Alternatively, there is the possibility that RYGB and SG produce comparable outcomes in food preferences through different mechanistic pathways. For example, consistent with clinical observations, findings from studies in rodent models show that both RYGB and SG reduce fat preferences [17,37,42,43,44] However, the decreased consumption of fat seems to be caused by a conditioned taste aversion in rodent models of SG [37] and by an increased sensitivity to fat reward in rodent models of RYGB [45]. In a recent series of elegant studies, Hankir and collaborators show that the rerouting of the intestine restored and enhanced intestinal synthesis of oleoylethanolamide, a lipid satiety factor and PPAR-α agonist that is reduced in obesity, which, via vagal activation, dramatically increased dorsal striatal dopamine signaling in response to high-fat food [45]. While intestinal rerouting enhances dopamine release in the dorsal striatum in response to fat, it reduces dopamine release in the dorsal striatum in response to sugars [46]. Glucose absorption through duodenum seems to be critical for striatal sugar-induced dopamine release and the stimulation of sweet appetite in the absence of hunger [46]. Whether or not the intestinal remodeling associated with delayed alimentary glucose absorption observed after SG [47] also blunts dorsal striatal sugar-induced dopamine release and sweet appetite is not known.

This study was limited due to the small number of participants, particularly in the SG group (*n* = 8). In addition, there was no separation by sex because, in agreement with the typical demographic distribution of the patients undergoing bariatric surgery [48], most of the subjects who participated in the study were women. For simplicity and design clarity, all taste stimuli used in the current study were presented in liquid form, but future studies should examine whether the findings are replicated when more ecologically relevant food stimuli (i.e., solid stimuli containing texture and smell in addition to taste) are used. This study examined the effects of short-term weight loss on eating behavior and taste perception. However, long-term studies are also needed as recent studies suggest the emergence of new eating disorders a few years after surgery [49,50]. Finally, the inclusion of a dietary management intervention post-surgery could have biased subjects’ hedonic responses to sweetness. However, the dietary management intervention was instrumental to control for potential important confounders, such as differences in weight loss between groups, of taste perception and eating behavior. Further, our previous findings that subjects who underwent LAGB did not experience altered hedonic responses to sweetness despite receiving the same dietary management intervention [20] suggest a bias of the dietary intervention on hedonic responses to sweetness is unlikely. 

## 5. Conclusions

Our results demonstrate that, despite the anatomical differences between RYGB and SG, weight loss induced by either procedure causes the same beneficial effects on key factors involved in the regulation of eating behavior and the hedonic component of taste perception. Future studies with larger samples should examine the alterations of the gut-brain axis by evaluating the biochemical (ghrelin, GLP-1, PYY) and neurological (mesolimbic dopamine reward pathway) factors in tandem with sensory tests. Progress in the identification of mechanisms underlying the many significant clinical changes induced by these surgeries may aid in the development of new knifeless alternatives to treat obesity and its metabolic disorders.

## Figures and Tables

**Table 1:** Subject Characteristics.

Variables	RYGB (*n* = 23)	SG (*n* = 8)	*p* Value
Age (years)	43.0 ± 9.6	36.6 ± 9.9	0.45
Race (%)			
White	78.3	75.0	
Black	13.0	25.0	0.54
Other/Mixed	8.7	0.0	
Gender (%)			
Female	87.0	87.5	0.97
Male	13.0	12.5	
Body Weight (kg)			
Before surgery	129.8 ± 26.1	143.3 ± 16.2	0.14
After surgery	104.6 ± 24.1	115.6 ± 12.8	0.20
% Weight Loss	19.8 ± 3.7	19.3 ± 1.8	0.77
Days to Achieve ~20% Weight Loss	99.2 ± 44.3	141.3 ± 27.7	0.02
BMI (kg/m^2^)			
Before surgery	46.9 ± 7.5	53.3 ± 8.7	0.31
After surgery	37.6 ± 6.7	43.0 ± 7.2	0.13

Values are %, mean ± standard deviation. Roux-en-Y Gastric Bypass; SG, Sleeve Gastrectomy BMI: Body mass index.

**Table 2:** Taste detection thresholds and scores on eating behavior questionnaires before and after ~20% body weight loss induced by RYGB or SG surgery.

Variable	RYGB (*n* = 23)		SG (*n* = 8)			
	Before Surgery	After Surgery	Before Surgery	After Surgery	*p* Value (Time)	*p* Value (Group X Time)
Detection Thresholds (mmol/L)						
Glucose	27.6 ± 14.1	27.6 ± 18.5	34.1 ± 5.3	39.5 ± 11.8	0.61	0.89
Sucrose	7.5 ± 5.1	6.5 ± 1.9	8.8 ± 5.0	6.1 ± 2.1	0.92	0.40
NaCl	2.4 ± 1.4	1.8 ± 0.9	2.0 ± 2.3	1.8 ± 1.1	0.24	0.66
MSG	1.2 ± 0.8	1.3 ± 0.9	1.3 ± 0.8	1.3 ± 1.3	0.89	0.46
Food Cravings						
High Fat	2.2 ± 0.7	1.8 ± 0.6	2.1 ± 0.6	1.6 ± 0.4	<0.01	0.78
Carbohydrates	2.2 ± 0.8	1.9 ± 0.7	2.6 ± 0.6	1.8 ± 0.5	<0.01	0.07
Sweets	2.3 ± 0.8	1.6 ± 0.6	2.5 ± 0.8	1.8 ± 0.6	<0.01	0.86
Fast Food	2.9 ± 0.7	2.2 ± 0.6	2.9 ± 0.7	2.0 ± 0.6	<0.01	0.40
DEBQ						
Restrained	2.9 ± 0.6	3.0 ± 0.7	2.9 ± 0.5	3.6 ± 0.6	0.02	0.06
Emotional	2.6 ± 0.9	1.8 ± 0.7	2.8 ± 0.9	2.2 ± 0.6	<0.01	0.57
External	3.0 ± 0.5	2.2 ± 0.5	3.1 ± 0.6	2.3 ± 0.3	<0.01	0.44

Values are mean ± standard deviation with the exception of detection threshold values that are median ± semi-interquartile range. RYGB, Roux-en-Y Gastric Bypass; SG, Sleeve Gastrectomy; DEBQ, Dutch Eating Behavior Questionnaire; NaCl, sodium chloride; MSG, monosodium glutamate.

## Supplementary Materials

The following are available online at http://www.mdpi.com/2072-6643/10/1/18/s1, Figure S1: Sucrose preferences, (a) Sucrose preferences before (white bars) and after (black bars) ~20% weight loss induced by bariatric surgery. Two-way ANOVA revealed a main effect of time (before surgery vs. after surgery) (*F*_(1,29)_ = 5.17; *p* = 0.03). RYGB and SG groups were combined because there was no main effect of group (*p* = 0.26) nor interaction (*p* = 0.64). Data are median values ± semi-interquartile range. **p* = 0.03. (b) Individual differences in sucrose concentrations preferred after surgery compared to sucrose concentrations preferred before surgery. RYGB, Roux-en-Y Gastric Bypass; SG, Sleeve Gastrectomy.
