# Supplementary material for: Effects of Sleeve Gastrectomy vs. Roux-en-Y Gastric Bypass on Eating Behavior and Sweet Taste Perception in Subjects with Obesity
Source: Nutrients. 2017 Dec 24;10(1):18. doi: 10.3390/nu10010018 (PMC5793246; doi:10.3390/nu10010018)
Supplement: Supplementary file 1 [file nutrients-10-00018-s001.pdf]

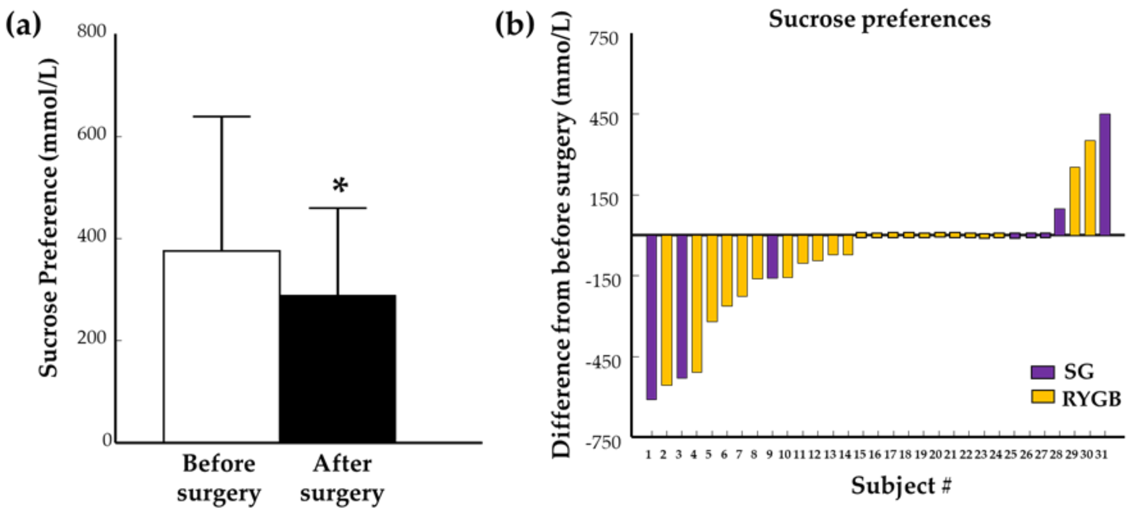

Figure S1: Sucrose preferences, (a) Sucrose preferences before (white bars) and after (black bars) ~20% weight loss induced by bariatric surgery. Two-way ANOVA revealed a main effect of time (before surgery vs. after surgery) ($F_{(1,29)}$=5.17; p=0.03). RYGB and SG groups were combined because there was no main effect of group (p=0.26) nor interaction (p=0.64). Data are median values ± semi-interquartile range. *p=0.03. (b) Individual differences in sucrose concentrations preferred after surgery compared to sucrose concentrations preferred before surgery. RYGB, Roux-en-Y Gastric Bypass; SG, Sleeve Gastrectomy.
